# Determinants of Plant Community Assembly in a Mosaic of Landscape Units in Central Amazonia: Ecological and Phylogenetic Perspectives

**DOI:** 10.1371/journal.pone.0045199

**Published:** 2012-09-18

**Authors:** María Natalia Umaña, Natalia Norden, Ángela Cano, Pablo R. Stevenson

**Affiliations:** 1 Universidad de Los Andes, Laboratorio de Ecología de Bosques Tropicales y de Primatología, Centro de Investigaciones Ecológicas La Macarena, Bogotá, Colombia; 2 Pontificia Universidad Javeriana, Departamento de Ecología y Territorio, Bogotá, Colombia; University of Helsinki, Finland

## Abstract

The Amazon harbours one of the richest ecosystems on Earth. Such diversity is likely to be promoted by plant specialization, associated with the occurrence of a mosaic of landscape units. Here, we integrate ecological and phylogenetic data at different spatial scales to assess the importance of habitat specialization in driving compositional and phylogenetic variation across the Amazonian forest. To do so, we evaluated patterns of floristic dissimilarity and phylogenetic turnover, habitat association and phylogenetic structure in three different landscape units occurring in *terra firme* (Hilly and Terrace) and flooded forests (Igapó). We established two 1-ha tree plots in each of these landscape units at the Caparú Biological Station, SW Colombia, and measured edaphic, topographic and light variables. At large spatial scales, *terra firme* forests exhibited higher levels of species diversity and phylodiversity than flooded forests. These two types of forests showed conspicuous differences in species and phylogenetic composition, suggesting that environmental sorting due to flood is important, and can go beyond the species level. At a local level, landscape units showed floristic divergence, driven both by geographical distance and by edaphic specialization. In terms of phylogenetic structure, Igapó forests showed phylogenetic clustering, whereas Hilly and Terrace forests showed phylogenetic evenness. Within plots, however, local communities did not show any particular trend. Overall, our findings suggest that flooded forests, characterized by stressful environments, impose limits to species occurrence, whereas *terra firme* forests, more environmentally heterogeneous, are likely to provide a wider range of ecological conditions and therefore to bear higher diversity. Thus, Amazonia should be considered as a mosaic of landscape units, where the strength of habitat association depends upon their environmental properties.

## Introduction

How tropical forests are able to harbour the Earth’s richest flora is one of the most challenging questions in community ecology. One possibility to explain such diversity is that tropical regions are mosaics of landscape units, promoting plant specialization to distinct habitat conditions [1], [2]. Such pattern has been reported in Western Amazonia [3], Panama [4], Borneo [5], the wet forests of Western Ghats in India [6], and in subtropical China [7]. Large-scale habitat heterogeneity is thus as an important driver of beta-diversity in tropical regions. At small spatial scales, community assembly is thought to be the result of local biotic interactions and environmental filtering [8], [9]. Yet, the importance of these processes is still debated as local floristic composition is also the result of dispersal from the regional species pool [10]–[14]. Since beta diversity provides a direct link between diversity at local and regional scales [15], determining the drivers of floristic dissimilarity across space may yield clues into how coexistence is maintained in tropical forests.

Regions characterized by mosaics of landscape units offer an excellent framework to address this issue, as local community structure may be driven by different processes in distinct landscape units. Phylogenetic-based analyses appear to be a compelling approach because it provides valuable information to disentangle among competing hypotheses, therefore offering a conceptual framework for the development of a synthetic ecological theory [16]–[18]. Here, we integrate information on ecological and phylogenetic data at different spatial scales to assess the importance of habitat specialization in driving compositional and phylogenetic variation in central Amazonia. Our approach takes advantage of the occurrence of a mosaic of patches of *terra firme* and flooded forests in the Colombian Amazon. In this region, a system of nutrient poor, black water flooded plains called Igapó, where trees are subject to long periods of flooding every year [19] is embedded in a landscape dominated by *terra firme* forests shaped by historical events occurring at different moments in space [20]. These distinct landscape units exhibit differences in species composition and structure, likely to be driven by edaphic factors [21]. If so, variation in the extent of floristic dissimilarity among sample units should mirror environmental differences among sites, independently of geographic distance. The examination of this issue usually relies on the comparison of species lists from forest inventories sampled along an environmental gradient. Yet, as phylogenetic relationships among species change across space, integrating phylogenetic turnover into these analyses further provides new insight elements to evaluate the degree of habitat association beyond the species level [22].

We expect different ecological processes to shape community structure in different landscape units, depending upon their abiotic properties. The stressful conditions found in Igapó are likely to sort species out, restricting the flora to species having particular adaptations to grow and persist in these demanding conditions [23]–[26]. Thus, Igapó is expected to show low tree diversity and phylodiversity, as well as the occurrence of species withstanding flood. As a result of such environmental filtering, and assuming that important traits show phylogenetic signal [13], then co-occurring species should be more related than expected by chance (i.e. phylogenetic clustering) [16], [17]. *Terra firme* forests, in contrast, show less physiological stress than Igapó, and a broader range of forest types underlying higher habitat heterogeneity. Defler & Defler [21] reported the occurrence of distinct physiographic units in *terra firme*, with areas of forest characterized by rolling hills dissected by brooklets (therein Hilly forests), and areas of different geomorphological history, suggesting past floodings during the Pleistocene (therein Terrace forests) [20]. Such heterogeneity at local and large scales may provide a wide range of ecological niches, allowing the coexistence of a higher number of species. Thus, we expect elevated levels of diversity and phylodiversity in *terra firme* forests, as well as an association between environmental factors and species occurrence. If local assemblages contain species with distinct ecological strategies in resource acquisition [27]–[29], and these strategies are phylogenetically conserved [13], then co-occurring species should be less related than expected by chance (i.e. phylogenic evenness) [16], [17].

To test these predictions, we collected information of six 1-ha plots in a lowland tropical forest in Vaupés, Colombia, comprising three major landscape units: one in flooded forests Igapó and two in *terra firme* forests (Terrace and Hilly forests) [21]. Specifically, we addressed the following questions: (1) Are diversity and phylodiversity lower in habitats subject to stressful environmental conditions? (2) To which extent do environmental differences across the landscape shape species and phylogenetic composition within and among landscape units? (3) Do local plant communities in flooded forests show phylogenetic clustering whereas those in *terra firme* phylogenetic evenness, and are these patterns conserved across spatial scales?

## Methods

### Study Site

This study was conducted at the Mosiro-Itajura Caparú Biological Station (CBS) (01°04′12′′S 069°30′55′′W), in the basin of the Apaporis river, Colombian Amazonia, where the average annual rainfall is 3950 mm and the mean annual temperature is 25°C [20]. Although there is no marked dry season (month <100 mm), the study area shows an annual flood pulse between March and October, caused by floods in the Apaporis river [20]. The station is dominated by pristine lowland forests growing in a geographically complex soil that combines acid and clayey soils from different geological ages [20]. According to edaphic, topographic and hydrological differences within the reserve, Defler & Defler [21] described five different landscape units: four on *terra firme* forests and one on floodplain forests. Here, we focused on the three most common: Hilly and Terrace forests (in *terra firme*), and Igapó (in floodplains). Hilly forests are characterized by small hills on clayey soils, Terrace forests are associated to areas that were flooded during the Pleistocene by the Apaporis river, and Igapó forests are flooded by black water for about eight months each year [20], [30].

### Data Colection

#### Tree censuses

Two 1-ha plots were established within each landscape unit. In each plot, all stems ≥10 cm diameter at breast height (DBH), including trees, palms and lianas, were tagged, and measured for DBH. Vouchers were collected from each stem, and identified to species or morpho-species at the ANDES and COAH herbaria in Bogotá, Colombia.

#### Abiotic variables

In all plots, the topographic profiles were measured within each 10×10 m quadrat using a clinometer (Suunto PM-5, USA). We collected soil samples in all 20×20 m quadrats for a total of 150 samples; each sample consisting in a mixture of topsoil (0 to 10 cm depth). These samples were subsequently analyzed for cation exchange capacity (CEC), clay, sand and silt percentage, and pH in a soil-analysis laboratory in Villavicencio, Colombia. We measured light intensity using a luxometer (Extech 407026, USA) at the center of each 20×20 m quadrat. To calibrate the measures made in the field, we took a reference measure in an open site, and the value for each point measured within the plots was expressed as the percentage of this reference point.

### Statistical Analyses

#### Floristic structure

At each plot, we evaluated species richness using species rarefaction curves, and species diversity using the Fisher’s alpha index [31]. Floristic similarity among plots was evaluated calculating the Chao-Jaccard estimator [32] with the package ‘vegan’ [33] in the R statistical software [34]. This estimator is an abundance-based similarity index that assesses the probability that individuals belong to shared *vs.* unshared species by accounting for the effect of unseen shared species. In tropical forests, where rare species are frequent and sampling is incomplete, this index is less biased by sample size, and thus more appropriate than other commonly used indices [32]. To characterize floristically each landscape unit, we calculated the Importance Value for each species by accounting by species relative frequency, density and dominance within each plot [35].

To evaluate the importance of environmental variables in determining floristic composition we performed two analyses. Because environmental similarity among plots was correlated with geographical distance (R_Mantel_  = 0.43 *P*<0.001), we first evaluated the extent to which environmental similarity accounted for species similarity, while controlling by geographical distance by using Partial Mantel Tests at different spatial scales: within plots, between plots within each landscape unit, and across landscape units. For all three scales, correlations among species similarity, environmental similarity and geographic distance were based on data from 20×20 m quadrats. Thus the spatial extent changed, while the resolution was kept constant. To reduce the multivariate environmental data, we performed a principal component analysis (PCA), and used the scores of the two principal components. The first PCA axis (PC1) was related to CEC and clay percentages in soil samples, and explained 35% of the variance in abiotic variables. The second one (PC2) was related to silt and sand percentages in soil samples, and explained 20% of the variance. Then, we performed a more specific analysis to test how floristic composition was associated to each of the environmental variables measured, by performing a canonical correspondence analysis (CCA). We tested for the significance of this association using an ANOVA like permutation test for CCA from the package ‘vegan’ [35] in the R statistical software [36]. The CCA analysis was performed at the 20×20 m scale.

#### Phylogenetic analyses

We constructed a phylogenic tree including all the species occurring in the study plots and those included in the list of the local flora (excluding shrubs and herbaceous plants) [36]. To do so, we used the angiosperm APGIII consensus tree (R20080417) [37], [38] from Phylomatic [41] as the backbone super-tree. This tree has family-level resolution, with most species and genera considered as polytomies within genera and families, respectively. Overall, we tagged 3526 individuals, of which 94% were identified to species and the remaining 6% to morpho-species. Since we were certain of the genus for all morpho-species, we included them in the phylogenetic tree. Branch lengths in the tree were adjusted to match clade age estimates reported by Wiksrom et al. [40] using the BLADJ algorithm. We performed several analyses based on this phylogenetic tree. First, we calculated the phylogenetic species richness of each plot using the phylogenetic species richness (PSR) index [41]. The PSR multiplies the number of species in the community by their evolutionary relatedness. This metric is related to the Faith’s phylogenetic diversity index, with the difference that the PSR uses more information contained within the phylogeny than does the Faith’s index [41]. Second, we evaluated the phylogenetic structure of the local assemblages at two spatial scales (landscape unit and plot level) using the phylogenetic species variability index (PSV) [41]. This metric indicates the degree to which co-occurring species are phylogenetically related to each other by measuring the among-species variance in the value of a hypothetical neutral trait evolving under a Brownian motion model. For a sample of *n* species,
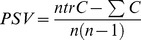



where C is the *n* x *n* sample phylogenetic covariance matrix, trC is the sum of diagonal elements of C and **Σ**C is the sum of all elements. As species in the sample become more closely related, the PSV decreases towards zero; and as species become less closely related, the PSV increased towards one; the statistical expectation of PSV is independent of species richness [41]. We also calculated phylogenetic species evenness, PSE, a formulation of the PSV that accounts for species abundance. Because both metrics showed similar trends, we only report the results obtained based on the PSV.The PSV was evaluated at the scale of the landscape unit and at the plot level. In the first case, this metric was calculated as the mean of the two plots within each landscape unit. At the plot level, this metric was calculated as the mean of the 25 20×20 m quadrats of each plot. These values were compared with a frequency distribution based in 1000 iterations generated using two null models: (1) the richness null model shuffles cells within each row so that the number of species within each community is preserved, but the prevalence of species changes across communities; (2) the frequency null model shuffles cells within each column so that the prevalence of each species is preserved but species richness within each community changes [41]. Because our a priori expectations predicted a specific phylogenetic pattern for each landscape unit (see Introduction), the observed means of PSV were compared to the 95% confidence intervals generated by the 1000 iterations based on one-tailed tests. As the presence of phylogenetic signal in species abundance can influence the patterns of phylogenetic structure observed, we tested whether abundant species were randomly distributed across the phylogeny by computing the ‘abundance phylogenetic deviation’ (APD) (see Table S1) [42]. This metric was not significantly different from zero, indicating that there was no phylogenetic signal in species abundance.

Finally, to evaluate the pairwise differences in species composition between communities incorporating phylogenetic information, we calculated the phylogenetic community dissimilarity (PCD) index [43]. This metric evaluates how much of the variance among species in the values of a hypothetical trait within a community can be predicted by the known trait values of species from another community [43]. This variance is calculated using the PSV index.
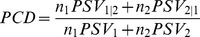
where PSV_1|2_ is calculated for community 2, conditional on information from community 1, and *n*_i_ is the number of species in the community *i* (for a more detailed description see Ives & Helmus [43]). If the PCD is greater than one, then communities tend to be phylogenetically dissimilar; and if the PCD is lower than one, then communities tend to be phylogenetically similar [43]. All the phylogenetic analyses were performed using the package ‘picante’ [44] in the R statistical software [42].

## Results

### Diversity and Descriptive Data

**Figure 1:**
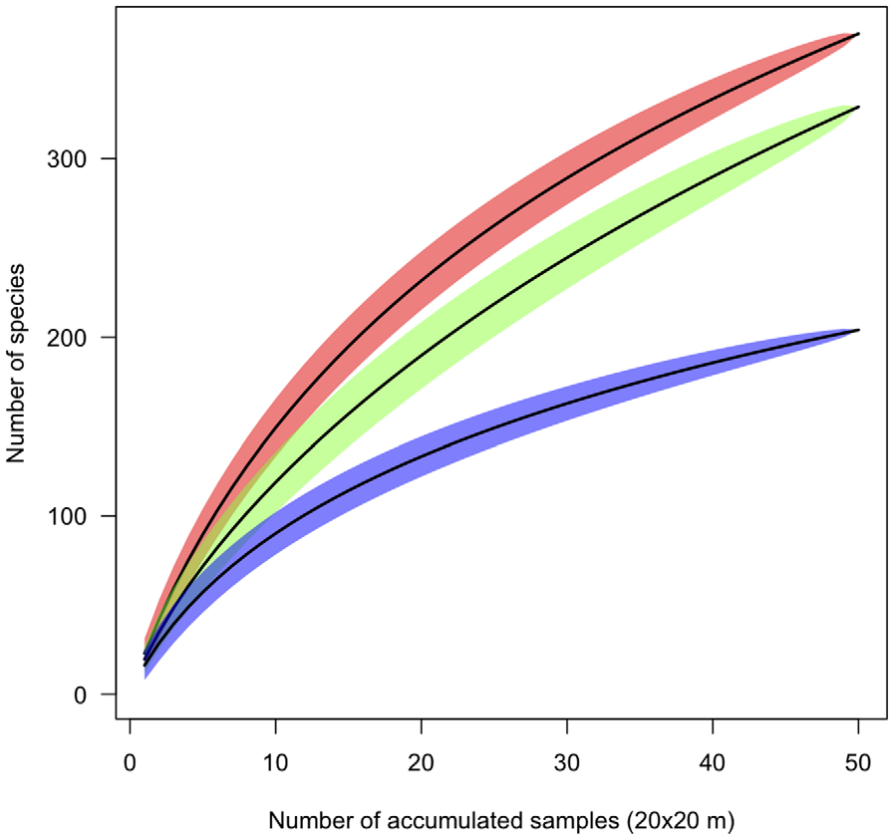
Species rarefaction curves within each landscape unit for trees >10 cm DBH. The red line denotes Hilly forests, the green line denotes Terrace forests and the blue line denotes Igapó forests. The shaded region represents 95% confidence intervals.

Terrace and Hilly forests showed similar stem density and species richness, while Igapó showed lower values in these two attributes (Table 1). Species rarefaction curves did not reach a saturation point; particularly in the *terra firme* plots (Fig. 1). The highest Fisher’s index was that of the Hilly forests, followed by Terrace and by Igapó. Patterns of phylogenetic diversity were in agreement with these trends: Igapó forests showed the lower values of PSR, followed by Terrace and by Hilly forests (Table 1).

**Table 1:** Number of individuals and diversity metrics based on trees ≥10 cm DBH, for each of the six 1-ha plots established at the Mosiro-Itajura Caparú Biological Station (Colombian Amazon).

Plot	Individuals	Species	Fisher’s Alpha	Observed PSR
Hilly 1	590	211	124.12	192.45
Hilly 2	641	256	155.27	166.31
Terrace 1	594	220	123.92	131.42
Terrace 2	634	171	78.36	168.60
Igapó 1	553	138	58.05	83.96
Igapó 2	514	116	47.75	103.98

Each landscape unit was characterized by different dominant species. Based on the Importance Value analysis, Hilly forests were dominated by four species: *Eschweilera coriacea* (Lecythidaceae), *Iryanthera ulei* (Myristicaceae), *Rinorea paniculata* (Violaceae) and *Euterpe precatoria* (Arecaceae). *Micrandra spruceana* (Euphorbiaceae) and *Oenocarpus bataua* (Arecaceae) showed the highest Importance Value in Terrace forests, and *Zygia cataractae* (Fabaceae) was an important species in Igapó forests. Table S2 summarizes the top 10 most important species in each of the six plots.

### Abiotic Variables

**Figure 2:**
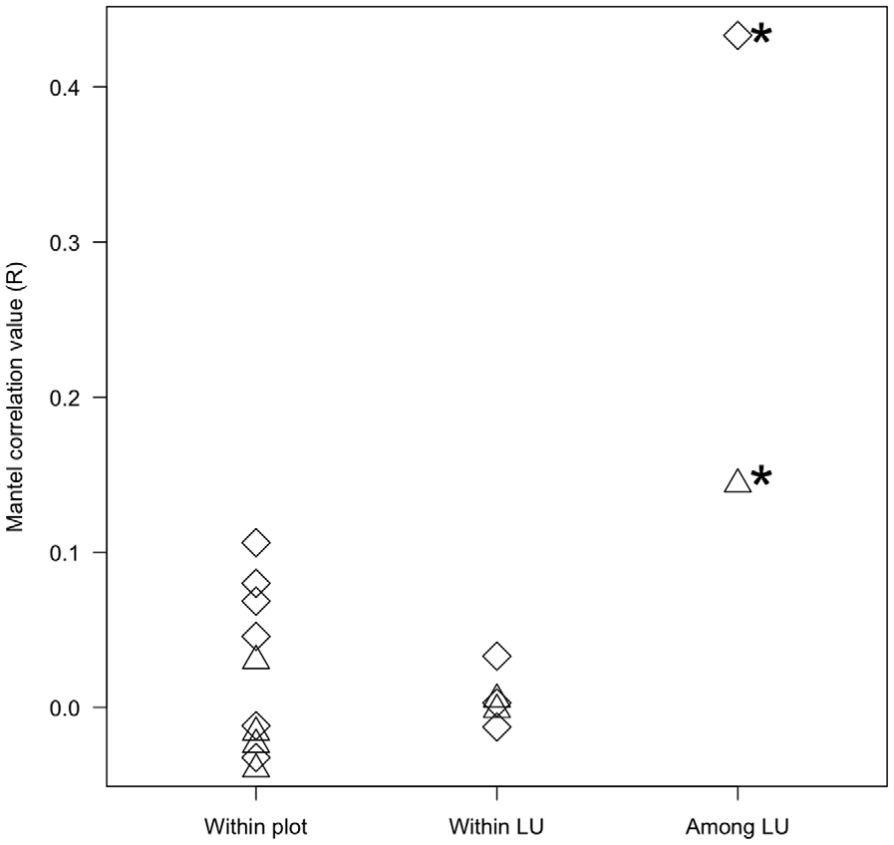
Correlation coefficients of Mantel test (R) relating floristic and environmental similarity, while controlling by distance, at three geographical scales: within plots, within landscape units (LU) and among landscape units. Open represent PC1 values and open represent PC2 values. *P*≤0.05 are indicated with asterisks.

**Figure 3:**
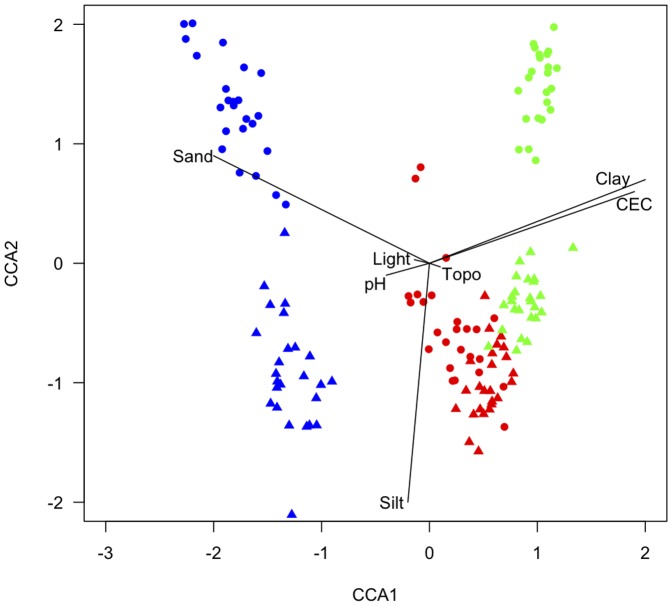
CCA of all tree species occurring in the six 1-ha plots. The arrows correspond to the abiotic variables included in the analysis. Symbols represent species of 20×20 m quadrants and show their association with the abiotic variables. Red triangles correspond to Hilly 1, red circles to Hilly 2; green triangles to Terrace 1, green circles to Terrace 2; blue triangles to Igapó forests plot 1, blue circles to Igapó plot 2.

**Figure 4:**
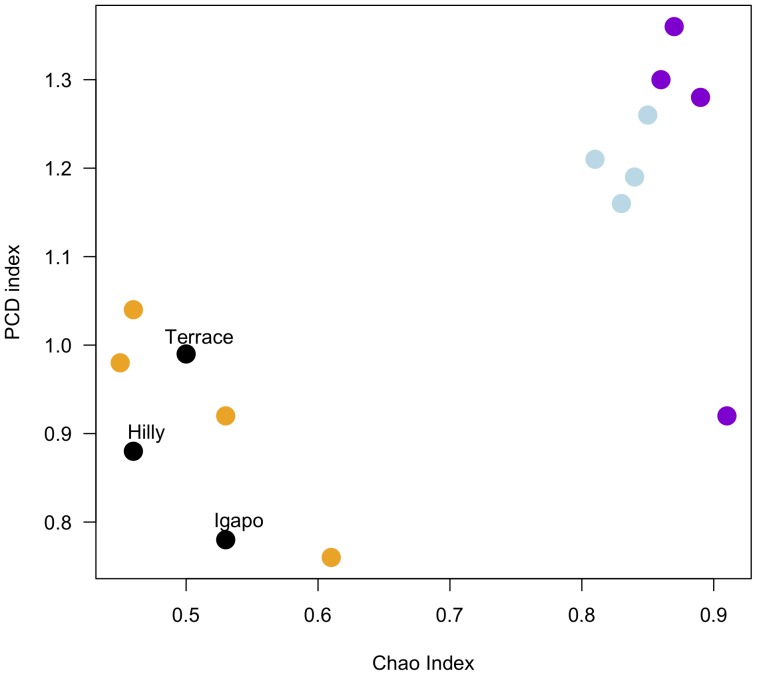
Pearson’s correlations between Chao and PCD indices. The orange dots represent coefficients calculated for Hilly and Terrace plots, blue dots represent coefficients calculated for Terrace and Igapó plots, and violet dots represent coefficients calculated for Igapó and Hilly plots. Black dots represent the coefficients for plots from the same landscape unit.

The partial Mantel partial tests revealed that floristic similarity within and between plots of the same landscape unit was not related to environmental factors. In contrast, at larger scales, floristic similarities among plots were significantly related to environmental factors (Fig. 2). Indeed, the CCA analysis showed that each landscape unit formed an independent floristic unit (axis 1∶22.9%, eigenvalue  = 0.67, axis 2∶14.3%, eigenvalue  = 0.42; Fig. 3). Igapó was the one exhibiting the most pronounced divergence in species composition (Fig. 3; Fig. 4). Silt and sand percentages, as well as CEC (Cation Exchange Capacity) and topography, were significantly correlated to the first two CCA axes (ANOVA: X^2^_Sand_ = 0.54, *P*_Sand_ = 0.01, F_ Sand_ = 2.86; X^2^_Silt_ = 0.46, *P*_Silt_ = 0.01, F_Silt_ = 2.46; X^2^_CEC_ = 0.22, *P*_CEC_ = 0.01, F_ CEC_ = 1.18; X^2^_Topo_ = 0.29, *P*_Topo_ = 0.01, F_Topo_ = 1.55; Fig. 3). The vectors most strongly correlated with species occurrence and relative abundance were the edaphic ones (Fig. 3). In particular, clay and CEC showed a strong correlation with many species in *terra firme* forests. Species composition between Terrace and Hilly forests was differentiated by the silt vector, indicating that Hilly forests grow over soils richer in silt. Soils of Igapó forests were the most infertile with low contents of silt and CEC. Light and pH were poorly related with species composition.

### Phylogenetic Structure

Phylogenetic community dissimilarity was lower between plots from the same landscape unit than between plots of different landscape units. The most dissimilar forests in terms of phylogenetic composition were Hilly and Igapó forests. Terrace forests were more similar to Hilly forests than to Igapó forests (Fig. 4).

**Figure 5:**
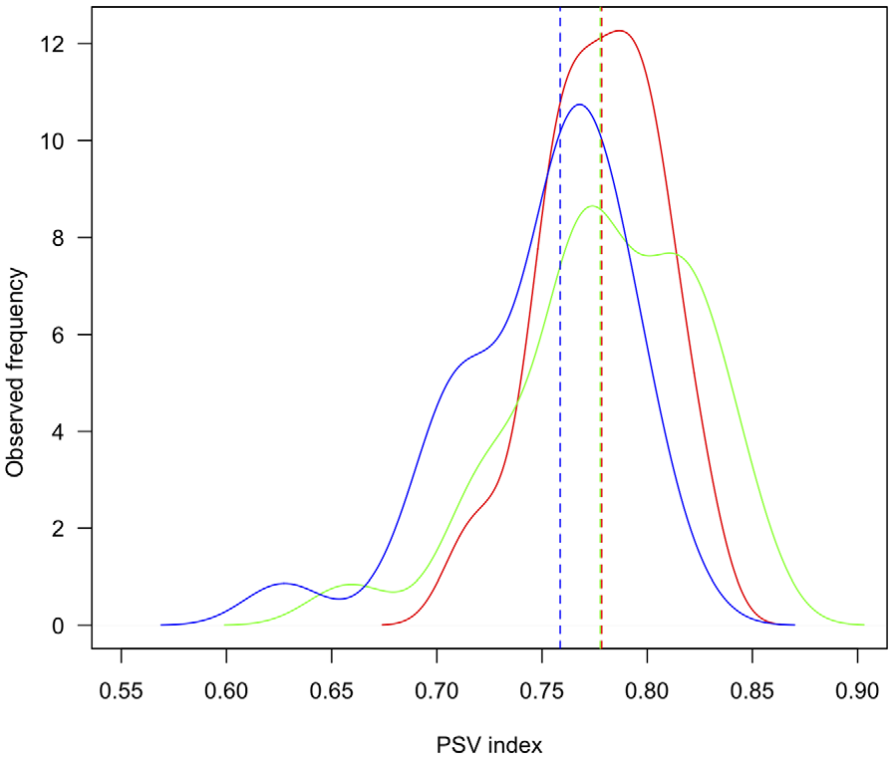
PSV values at 20×20 m scale for each landscape unit. The red line corresponds to Hilly forests, the green line to Terrace forests and the blue line to Igapó forests. The dashed line represents the median of the distribution for each landscape unit.

As expected, flooded forests showed a phylogenetic clustering whereas *terra firme* forests showed phylogenetic evenness (Table 2). More specifically, in Igapó forests, mean PSV was lower than expected using both null models, but this result was significant only with the frequency null model (PVS_FN_). In Hilly forests, mean PSV was higher than expected using both null models, but again, this result was significant only with PVS_FN_. Finally, in Terrace forests, mean PSV was higher than expected using both null models but significantly so only with the richness null model (PVS_RN_) (Table 2). At the plot level, PSV values were not significantly different from zero in most of the cases, but exhibited similar trends as those found at the landscape unit level, with Igapó showing lower values than Hilly and Terrace forests (Fig. 5; Table S3).

**Table 2:** Phylogenetic species diversity (PSV) for each landscape unit.

Plot	Null model	Observed PSV	Confidence intervals of the randomized PSV
**Hilly**	Richness	0.758	0.749–0.768
**Hilly**	Frequency	**0.758**	0.756–0.757
**Terrace**	Richness	**0.774**	0.749–0.773
**Terrace**	Frequency	0.774	0.773–0.774
**Igapó**	Richness	0.759	0.746–0.774
**Igapó**	Frequency	**0.759**	0.761–0.763

Significant values (*P*≤0.05, one-tailed test) are indicated in bold. Based on our a priori hypotheses, for Hilly and Terrace forests, PSV scores are significant if higher than the 95% quantile of randomized PSV. For Igapó forests, PSV scores are significant if lower than the 5% quantile of randomized PSV.

## Discussion

Patterns of diversity and species composition showed important variation among landscape units, particularly between flooded and *terra firme* forests. In agreement with previous studies conducted in the Amazonia [22], [30], [45], *terra firme* exhibited higher levels of diversity than Igapó, and relatively few species were shared between these two types of forests (13% and 20% between Igapó-Hilly and Igapó-Terrace forests, respectively). Such floristic dissimilarity is accompanied by a phylogenetic divergence, suggesting that the sorting due to flood goes beyond the species level. These patterns have been previously reported in SE Asia, were different families were associated with distinct habitats [22]. If so, our results suggest that the low diversity observed in Igapó, and the compositional and phylogenetic differences observed between flooded and non-flooded systems are the outcome of habitat specialization. This pattern, however, is not necessarily driven by differences in competitive abilities among species occurring in distinct habitats. For instance, Fine et al. [46], showed that habitat association patterns in clayey and sandy forests in the Peruvian Amazonia was mediated by differences in antiherbivore defenses among species.

Within *terra firme,* Hilly and Terrace forests shared an important fraction of species (53%), suggesting that a large extent of the floristic divergence observed between these two landscape units relied on species abundance rather that on species incidence. For instance, *O. bataua* and *E. coriacea* occurred in all *terra firme* plots but were more abundant in Terrace and Hilly forests, respectively. These species are known to be generalists, persisting in a wide range of environments across the Amazonia [47], and probably exhibiting a broad range of environmental tolerances. This explains the high similarity between one of the plots in Terrace forests and the Hilly forests plots. However, each landscape unit did exhibit independent floristic units. For instance, *M. spruceana* was particularly dominant in Terrace forests, but was totally absent in Hilly forests. Together, these findings demonstrate that species relative abundance and distribution varies not only between flooded and non-flooded systems but also within *terra firme* forests. Yet, these differences are not reflected by phylogenetic similarity analyses. The PCD index comparing phylogenetic relatedness between Hilly and Terrace forests exhibited values close to one, indicating that, phylogenetically, these stands are not significantly different from communities selected at random from the species pool [43].

Within landscape units, comparing patterns of floristic dissimilarity and phylogenetic turnover brought insightful elements to understand local community assembly at more local scales. For example, Igapó showed the highest floristic divergence between plots of the same landscape unit, but also showed low phylogenetic turnover. Because these two plots are located at the two opposite shores of the Apaporis river, dispersal limitation may be, in part, the cause of such floristic divergence. Alternatively, as the river stream does not exert the same lateral erosive process at each side, differences in sedimentation, nutrient depletion and deposition might have affected the successional process occurring at each of these locations [48]. Floristic divergence may therefore be the outcome of different successional stages resulting from perturbations that occurred at distinct moments. Finally, it could be the outcome of alternative trajectories that reached different stable states [49]. In any of these scenarios, our findings suggest that Igapó forests are subject to constant disturbance due to flood.

Overall, our findings indicate that each landscape unit harbors relatively different plant communities. The correlation between floristic similarity and geographic distance suggests that differences in species composition among landscape units are the outcome of dispersal limitation. Poor dispersal has been widely reported for tropical trees [50], [51] suggesting that spatial processes are important in determining the local abundance of many species [52], [53]. Yet, these conclusions need to be taken with caution, as environmental similarity was tightly correlated with geographical distance. Indeed, our findings may also by the result of a spurious effect arising from the geographic location of the landscape units. The distance between the plots established in Igapó, the landscape unit showing the most conspicuous differences in species composition with the other two, is longer than the distance between the plots of Terrace and Hilly forests. We would need a more extensive sampling in a wider geographical range to address this issue more straightforwardly.

We found that floristic and environmental similarity were significantly correlated only at large scales, indicating that the steeper the gradient in environmental variation, the stronger the influence of environment in species composition [54]. Similar findings have been found in white sand [18] and flooded forests [55] in the Peruvian Amazon, and in Panama [56]. Indeed, many studies have documented habitat association driven by physical factors, in particular by soil variables [2], [3], [57], [58]. Our description of edaphic conditions within each landscape unit showed that the marked differences in floristic composition observed among landscape units were strongly associated with soil characteristics, differentiated by contents of sand, clay and silt. Although these results are globally in agreement with Defler & Defler [21], we found some discrepancies between their study and ours regarding Igapó’s edaphic composition. Specifically, we found that Igapó was the sandiest landscape unit, whereas Defler & Defler [21] found very high contents of clay. Because their analyses were based on a low number of replicates within each forest type, they might have overlooked the whole variation in soil composition exhibited within each landscape unit.

Among the other environmental variables studied, only topography seemed to have an effect on species composition in Hilly forests. Similar pH values were found within and across plots, indicating that this factor was irrelevant to discriminate among landscape units. Finally, light availability also appeared to be poorly correlated with species composition. Because *terra firme* forests harbor higher stem density than Igapó forests, one would have expected shade-tolerant species to be associated with the limiting light conditions in the forest. Yet, such association was not found because light availability did not show strong variation among landscape units. Moreover, light is a limiting factor for plant growth and establishment particularly during early stages [59], [60], but at adult stages, it is difficult to detect the footprint of a process that occurred long time ago.

The habitat association patterns observed may be the outcome of different niche-based processes. As predicted, Igapó showed phylogenetic clustering but only under the frequency null model, suggesting that the strong relatedness found among co-occurring species in this landscape unit is driven by nonrandom associations between species among communities [41]. Following, the seminal ideas developed by Webb et al. [16], these results suggest a major role of environmental filtering. Recent findings have highlighted that phylogenetic clustering may also be driven by competition [61]. However, in the light of our results, we believe that community assembly in Igapó is strongly governed by the environmental stress imposed by flooding. At local scales, species did not show any particular trend. This is not surprising since environmental filtering is typically more conspicuous at large spatial scales [62], [63].

Conversely, both landscape units in *terra firme* forests showed phylogenetic evenness. Hilly forests showed a significant pattern under the frequency null model, whereas Terrace forests did so under the richness null model. These results suggest that evenness in Hilly forests is driven by nonrandom associations between species among communities, whereas in Terrace forests it is driven by differences in the overall prevalence of species [41]. Together, these findings would suggest that biotic interactions play a major role in structuring plant communities in *terra firme* forests [16], [17]. Yet, at the plot level, most plant assemblages did not show any phylogenetic structure. These results weaken the role of competition in structuring *terra firme* forests, as this ecological process operates at local scales. Because both Hilly and Terrace forests exhibited higher variation in topography (SD = 2.14 and 1.79, respectively) compared to Igapó forests (SD = 0.81), we believe that the observed pattern of phylogenetic evenness may reflect niche differentiation rather than competition. Topography may stand as a proxy of soil resources not measured in this study, such as water availability and drainage, suggesting that forests in *terra firme* might offer a wider diversity of niches, allowing the establishment of species with broad ecological strategies [64]. Also, negative density dependent processes may lead to an even phylogenetic structure, if plant enemies reduce the establishment of individuals closely related to focal species [65]. Yet, this process is more likely to occur at early stages, when negative density dependence is more intense [66].

The recent flourishing of phylogenetic methods has allowed the reappraisal of classical ecological theories. Here we integrate ecological and evolutionary approaches to evaluate the importance of environmental factors in shaping community assembly in a mosaic of landscape units. Edaphic specialization was an important driver of floristic and phylogenetic distinctiveness across the landscape, whereas the role of competition appeared to be weak.

Further understanding of the processes shaping community structure within each landscape unit needs a functional perspective. In particular, root and seed traits may be good indicators of plant ability to establish and prevail in flooded plains [24], [25]. Likewise, maximum height could help to understand the role of local biotic interactions in both flooded and non-flooded forests [67].

## Supporting Information

Table S1 APD values for each plot.
(DOCX)

Table S2 List of the 10 most important species according to Importance Value.
(DOCX)

Table S3 Phylogenetic Species Variability (PSV) within each 1-ha plot.
(DOCX)
