# Supplementary material for: Determinants of Plant Community Assembly in a Mosaic of Landscape Units in Central Amazonia: Ecological and Phylogenetic Perspectives
Source: PLoS One. 2012 Sep 18;7(9):e45199. doi: 10.1371/journal.pone.0045199 (PMC3445462; doi:10.1371/journal.pone.0045199)
Supplement: Table S1 — APD values for each plot. (DOCX) [file pone.0045199.s001.docx]

**Table S1. APD values for each plot.** SD means standard deviation, SES means standardized effect size and it is calculated (obs – Mean Null)/ SD. Null distribution and quintile represents the rank of observed values in the null distribution (N = 1000).

|  | **Observed** | **Mean Null** | **SD Null Distribution** | **SES** | **Quantile** |
| --- | --- | --- | --- | --- | --- |
| **Hilly 1** | 0.008 | 0.012 | 0.016 | -0.289 | 339 |
| **Hilly 2** | -0.015 | 0.012 | 0.017 | -1.575 | 70 |
| **Terrace 1** | 0.006 | 0.046 | 0.033 | -1.211 | 165 |
| **Terrace 2** | 0.043 | 0.025 | 0.025 | 0.705 | 738 |
| **Igapó 1** | 0.059 | 0.029 | 0.026 | 1.144 | 858 |
| **Igapó 2** | 0.058 | 0.034 | 0.028 | 0.871 | 798 |
