# Supplementary material for: Determinants of Plant Community Assembly in a Mosaic of Landscape Units in Central Amazonia: Ecological and Phylogenetic Perspectives
Source: PLoS One. 2012 Sep 18;7(9):e45199. doi: 10.1371/journal.pone.0045199 (PMC3445462; doi:10.1371/journal.pone.0045199)
Supplement: Table S2 — List of the 10 most important species according to Importance Value. (DOCX) [file pone.0045199.s002.docx]

**Table S2. List of the 10 most important species according to the Importance Value**.

Abbreviations: IV: Importance Value [35] in each of the six 1-ha plots.

| **Species Hilly 1** | **Density** | **Freqency** | **Dominance** | **IV** |
| --- | --- | --- | --- | --- |
| *Eschweilera coriacea* | 6.13 | 76 | 4.2 | 86.33 |
| *Virola carinata* | 2.32 | 48 | 1.65 | 51.97 |
| *Iryanthera ulei* | 3.15 | 52 | 0.78 | 46.93 |
| *Rinorea paniculata* | 3.15 | 40 | 0.69 | 43.84 |
| *Scleronema praecox* | 1.66 | 40 | 0.99 | 42.64 |
| *Cynometra longicuspis* | 1.82 | 36 | 4.19 | 42.01 |
| *Monopteryx uaucu* | 2.32 | 32 | 7.11 | 41.43 |
| *Iryanthera laevis* | 2.15 | 44 | 2.49 | 39.98 |
| *Iryanthera juruensis* | 1.49 | 36 | 0.35 | 37.84 |
| *Euterpe precatoria* | 1.82 | 32 | 0.43 | 34.25 |

| **Species Hilly 2** | **Density** | **Freqency** | **Dominance** | **IV** |
| --- | --- | --- | --- | --- |
| *Eschweilera coriacea* | 6.6 | 88 | 7.5 | 102.1 |
| *Protium* cf *aracouchini* | 1.3 | 48 | 2.5 | 51.8 |
| *Iryanthera ulei* | 0.7 | 40 | 1.9 | 42.6 |
| *Micropholis venulosa* | 1.8 | 36 | 1.6 | 39.4 |
| *Iriartea deltoidea* | 1.2 | 36 | 1.7 | 39.0 |
| *Brosimum lactescens* | 1.9 | 32 | 1.3 | 35.2 |
| *Clathrotropis macrocarpa* | 1.1 | 32 | 1.9 | 35.0 |
| *Rinorea paniculata* | 1.2 | 32 | 1.7 | 34.9 |
| *Pseudolmedia laevis* | 0.9 | 32 | 1.2 | 34.1 |
| *Euterpe precatoria* | 0.6 | 32 | 1.6 | 34.1 |
|  |  |  |  |  |
| **Species Terrace 1** | **Density** | **Frequency** | **Dominance** | **IV** |
| *Micrandra spruceana* | 10.82 | 96 | 14.9 | 121.72 |
| *Eschweilera coriacea* | 6.99 | 76 | 3.5 | 86.49 |
| *Pseudosenefeldera inclinata* | 4.33 | 60 | 1.54 | 65.86 |
| *Clathrotropis macrocarpa* | 3.16 | 48 | 1.24 | 52.4 |
| *Hevea guianensis* | 2.33 | 48 | 2.04 | 52.37 |
| *Sandwithia heterocalyx* | 2.5 | 40 | 0.56 | 43.06 |
| *Sorocea muricata* | 2 | 40 | 0.8 | 42.8 |
| *Monopteryx uaucu* | 1.66 | 32 | 9.09 | 42.75 |
| *Endlicheria formosa* | 1.83 | 40 | 0.57 | 42.4 |
| *Dacryodes belemensis* | 1.33 | 32 | 0.56 | 33.89 |

| **Species Terrace 2** | **Density** | **Freqency** | **Dominance** | **IV** |
| --- | --- | --- | --- | --- |
| *Oenocarpus bataua* | 8.2 | 100 | 18.1 | 126.3 |
| *Micrandra spruceana* | 10.3 | 76 | 5.0 | 91.3 |
| *Monopteryx uaucu* | 14.9 | 56 | 3.0 | 73.9 |
| *Euterpe precatoria* | 1.1 | 60 | 4.6 | 65.7 |
| *Caraipa punctulata* | 2.2 | 48 | 2.7 | 52.8 |
| *Brosimum rubescens* | 3.9 | 44 | 2.5 | 50.4 |
| *Protium* cf *aracouchini* | 1.7 | 44 | 2.7 | 48.4 |
| *Sandwithia heterocalyx* | 0.9 | 44 | 3.2 | 48.1 |
| *Iryanthera ulei* | 0.7 | 44 | 2.2 | 46.9 |
| *Virola pavonis* | 2.4 | 40 | 2.2 | 44.6 |

| **Species Igapó 1** | **Density** | **Frequency** | **Dominancia** | **IV** |
| --- | --- | --- | --- | --- |
| *Aldina heterophylla* | 8.14 | 80 | 7.95 | 96.09 |
| *Zygia cataractae* | 7.96 | 76 | 5.34 | 89.3 |
| *Eschweilera albiflora* | 3.98 | 60 | 9.55 | 73.53 |
| *Dicorynia paraensis* | 4.16 | 60 | 7.56 | 71.72 |
| *Eschweilera integrifolia* | 3.44 | 60 | 1.94 | 65.38 |
| *Licania egleri* | 3.8 | 52 | 1.57 | 57.36 |
| *Didymocistus chrysadenius* | 5.06 | 48 | 3.98 | 57.04 |
| *Manilkara bidentata* | 3.62 | 44 | 2.43 | 50.05 |
| *Cynometra longicuspis* | 2.89 | 40 | 1.95 | 44.84 |
| *Virola elongata* | 3.07 | 36 | 1.38 | 40.45 |

| **Species Igapó 2** | **Density** | **Freqency** | **Dominance** | **IV** |
| --- | --- | --- | --- | --- |
| *Caraipa densifolia* | 8.8 | 68 | 12.3 | 89.1 |
| *Zygia cataractae* | 2.8 | 56 | 4.9 | 63.7 |
| *Aldina heterophylla* | 3.8 | 44 | 3.1 | 50.9 |
| *Virola elongata* | 1.4 | 44 | 4.9 | 50.3 |
| *Dicorynia* cf *paraensis* | 6.7 | 36 | 3.3 | 46.0 |
| *Acosmium nitens* | 4.6 | 36 | 2.1 | 42.7 |
| *Buchenavia viridiflora* | 7.3 | 32 | 3.1 | 42.4 |
| *Aspidosperma excelsum* | 2.5 | 36 | 3.3 | 41.8 |
| *Cynometra marginata* | 2.0 | 36 | 2.7 | 40.7 |
| *Crudia oblonga* | 1.7 | 36 | 2.5 | 40.2 |
