# Supplementary material for: Determinants of Plant Community Assembly in a Mosaic of Landscape Units in Central Amazonia: Ecological and Phylogenetic Perspectives
Source: PLoS One. 2012 Sep 18;7(9):e45199. doi: 10.1371/journal.pone.0045199 (PMC3445462; doi:10.1371/journal.pone.0045199)
Supplement: Table S3 — Phylogenetic Species Variability (PSV) within each 1-ha plot. (DOCX) [file pone.0045199.s003.docx]

**Table S3.** **Phylogenetic Species Variability (PSV) within each 1-ha plot.** The PSV values are an average of the 25 subplots within 1-ha plot. Significant values (*P* ≤ 0.05, one-tailed test) are indicated in bold.

| **Plot** | **Null model** | **Observed PSV** | **PSV null (C.I.)** | **Phylostructure** |
| --- | --- | --- | --- | --- |
| **Hilly 1** | Frequency | 0.773 | 0.774 (0.770-0.777) | Random |
|  | Richness | 0.773 | 0.761 (0.749-0.773) | Random |
| **Hilly 2** | Frequency | 0.788 | 0.787 (0.783-0.790) | Random |
|  | Richness | **0.788** | 0.759 (0.745-0.774) | Even |
| **Terrace 1** | Frequency | 0.797 | 0.795 (0.792-0.798) | Random |
|  | Richness | **0.797** | 0.761 (0.745-0.775) | Even |
| **Terrace 2** | Frequency | 0.766 | 0.766 (0.762-0.769) | Random |
|  | Richness | 0.766 | 0.760 (0.745-0.775) | Random |
| **Igapó 1** | Frequency | **0.741** | 0.735 (0.729-0.740) | Even |
|  | Richness | **0.741** | 0.760 (0.745-0.775) | Clustered |
| **Igapó 2** | Frequency | 0.758 | 0.759 (0.753-0.764) | Random |
|  | Richness | 0.758 | 0.761 (0.748-0.773) | Random |
